# Recombinant expression of *Streptococcus pneumoniae* capsular polysaccharides in *Escherichia coli*

**DOI:** 10.1098/rsob.150243

**Published:** 2016-04-13

**Authors:** Emily J. Kay, Laura E. Yates, Vanessa S. Terra, Jon Cuccui, Brendan W. Wren

**Affiliations:** Department of Pathogen Molecular Biology, London School of Hygiene and Tropical Medicine, Keppel Street, London WC1E 7HT, UK

**Keywords:** *Streptococcus pneumoniae*, synthetic glycobiology, glycoengineering, vaccine, capsular polysaccharide

## Abstract

Currently, *Streptococcus pneumoniae* is responsible for over 14 million cases of pneumonia worldwide annually, and over 1 million deaths, the majority of them children. The major determinant for pathogenesis is a polysaccharide capsule that is variable and is used to distinguish strains based on their serotype. The capsule forms the basis of the pneumococcal polysaccharide vaccine (PPV23) that contains purified capsular polysaccharide from 23 serotypes, and the pneumococcal conjugate vaccine (PCV13), containing 13 common serotypes conjugated to CRM197 (mutant diphtheria toxin). Purified capsule from *S. pneumoniae* is required for pneumococcal conjugate vaccine production, and costs can be prohibitively high, limiting accessibility of the vaccine in low-income countries. In this study, we demonstrate the recombinant expression of the capsule-encoding locus from four different serotypes of *S. pneumoniae* within *Escherichia coli*. Furthermore, we attempt to identify the minimum set of genes necessary to reliably and efficiently express these capsules heterologously. These *E. coli* strains could be used to produce a supply of *S. pneumoniae* serotype-specific capsules without the need to culture pathogenic bacteria. Additionally, these strains could be applied to synthetic glycobiological applications: recombinant vaccine production using *E. coli* outer membrane vesicles or coupling to proteins using protein glycan coupling technology.

## 1. Background

*Streptococcus pneumoniae* is an important human pathogen worldwide, with an estimated 14 million cases and 1 million deaths annually, particularly among young children [1]. Although nasopharyngeal colonization is common, *S. pneumoniae* can also cause invasive disease. Pneumonia is the primary outcome, but secondary infections can result in meningitis, bacteraemia and otitis media [1].

There are 95 serotypes of *S. pneumoniae* currently recognized, in a scheme based on the cell surface located capsular polysaccharide [2–6]. Some serotypes are more often associated with invasive disease. Serotype-specific odds ratios for invasive disease showed that serotypes 3, 6A and 15 are often found in carriage, whereas serotypes 1, 5 and 7 are usually associated with invasive disease in children [7]. Serotype prevalence can change according to geographical region and age of those infected. A review of more than 70 studies found that serotypes 1, 6, 14, 19 and 23 are prominent across all continents [8]. In addition, certain serotypes are more commonly associated with a particular disease outcome. A surveillance study in Germany 1992–2013 found pneumococcal meningitis cases were associated with serotypes 14, 7F, 3, 19F and 23F [9], whereas in the African meningitis belt pneumococcal meningitis is predominantly caused by serotype 1 [10]. Isolates with the same multilocus sequence type but different serotypes have different invasive potential, which suggests that capsule may be more important than genotype with regards to invasive disease [11]. Analysis of capsule-switched strains indicated that the capsule is the major determinant of resistance of the strain to complement and opsonophagocytosis [12]. Serotype 19F is highly resistant to non-opsonic phagocytosis [12], and higher levels of anti-capsular antibodies are required for killing [13] and therefore protection against disease caused by this serotype. Capsule type influences growth dynamics; those able to produce thicker capsules at a lower metabolic cost are associated with higher prevalence in carriage [14,15].

The capsule of *S. pneumoniae* is a major immunogen, so the majority of vaccines are based on purified capsular polysaccharide, or polysaccharide conjugated to an immunogenic protein. Currently licensed vaccines include the pneumococcal conjugate vaccine (PCV13) and the pneumococcal polysaccharide vaccine (PPV23). PPV contains 23 capsular types, 1, 2, 3, 4, 5, 6B, 7F, 8, 9N, 9 V, 10A, 11A, 12F, 14, 15B, 17F, 18C, 19F, 19A, 20, 22F, 23F and 33F, and is recommended for people over 65 or with underlying health conditions. PPV23 has been shown to have a limited effect on carriage [16], and it also produces a T-cell-independent response so is not effective in children under 2 years of age. Prevnar PCV13 contains serotypes: 1, 3, 4, 5, 6A, 6B, 7F, 9 V, 14, 18C, 19F, 19A and 23F conjugated to a deactivated diphtheria toxin protein, CRM197 [17]. Conjugation of a polysaccharide to a protein carrier allows presentation by B-cells to T-cells allowing for mucosal immunity and immunological memory [18].

Currently, to synthesize conjugate vaccines, each reaction requires optimization as each polysaccharide is chemically distinct. Multiple stages and rounds of purification are necessary, so yields of the final product are low [19]. Capsular polysaccharide must be purified from the pathogen while avoiding contamination from other bacterial polysaccharides. The polysaccharide is then activated and chemically linked to a protein. The chemical activation used may alter the conformation and epitopes presented, and the conjugates produced are heterologous in terms of linkage position. Finally, the resulting conjugate must be purified to remove unconjugated protein and polysaccharide [19]. More recently, it has been shown that recombinant glycoconjugate vaccines can be produced in *Escherichia coli* where the glycan (e.g. polysaccharide capsule) is coupled to a carrier protein by a bacterial oligosaccharyltransferase, PglB [20]. This process has been termed protein glycan coupling technology (PGCT) and has recently been used to produce vaccines against a range of pathogens [21–24]. In order for PGCT to be used to create novel *S. pneumoniae* vaccines, first the capsular polysaccharide must be expressed within *E. coli*.

There are two methods of capsule assembly in *S. pneumoniae*: first, a synthase-dependent reaction whereby sugars are assembled in a sequential fashion processively; second, a polymerase-dependent reaction whereby repeat units are linked [25]. Only two *S. pneumoniae* capsule structures use the synthase method: serotypes 3 and 37. The serotype 3 capsule has been assembled in *E. coli* on a lipid primer by expressing the synthase gene and using sugar precursors from *E. coli* [26]. In the polymerase-dependent reaction, an initial transferase attaches a nucleotide-activated sugar to a membrane-bound undecaprenyl pyrophosphate (Und-PP) carrier. Specific transferases then attach further sugars in turn until a complete subunit has been formed. This subunit is then translocated across the membrane by a flippase, after which a polymerase enzyme links the subunits at specific sites. The polymerized CPS is attached to the cell wall by a complex of Wzd/Wze, and the undecaprenyl pyrophosphate carrier is released to be used again [25].

The structure of each capsule varies, containing different combinations produced from 21 components identified so far. The genes for synthesis and assembly of the capsule are usually encoded on a contiguous DNA fragment between *dexB* and *aliA*, neither of which have a function in capsule biosynthesis [27]. The sequence of 90 capsule coding regions has been published [28] and ranges in size from 10 to 30 kb. Of the 95 serotypes, 54 have some structural data, for the remainder there is no structural data although for some a component sugar analysis has been reported [28,29]. Of the 21 known components found in the capsule structures, 13 are produced via enzymes encoded on the capsule locus, and eight are supplied by housekeeping pathways. The eight components supplied by *S. pneumoniae* pathways contained outside of the capsule locus are: UDP-glucose, UDP-galactose, UDP-GlcNAc, UDP-GalNAc, pyruvate, CDP-choline, CDP-ribitol and UDP-AATGal [30]. It is likely that the first five could be supplied by *E. coli* central metabolism, but the remainder would require heterologous expression of further enzymes [28,30].

In this study, we clone and express selected capsular polysaccharide serotypes in *E. coli* with a view to fully understand the role of all genes required to express a given serotype, and to use the recombinant capsule as a platform for vaccine development. Cloning into *E. coli* allows for dissection of the features that constitute the pathway in a more tractable system. In general, the expression of complex glycans in *E. coli* will facilitate synthetic glycobiological approaches that are increasingly required in biotechnological applications including vaccine design. We report the expression of the capsular polysaccharide of serotypes 4, 5, 8 and 12F. In addition, this study provides insights into rational design for recombinant expression and which further serotypes would be tractable for recombinant expression.

## 2. Methods

### 2.1. Bacterial strains and plasmids

*Escherichia coli* strains were cultured on modified super optimal broth (SSOB) agar at 28°C, or grown for 16 h in SSOB at 28°C. Where necessary, antibiotics were added: tetracycline (20 µg ml^−1^) and trimethoprim (100 µg ml^−1^; see electronic supplementary material, table S1 for strains and plasmids used in this study). *S. pneumoniae* were grown for 16 h at 37°C on brain heart infusion (BHI) agar with 5% horse blood, or statically in BHI broth, in an atmosphere containing 5% CO_2_.

### 2.2. Synthesis, PCR and cloning of capsule loci

All capsule loci were either synthesized commercially (serotype 4: Epoch Life Sciences; serotype 5: GeneWiz; serotype 12F: GenScript) or amplified by PCR (serotype 8). Genomic DNA from *S. pneumoniae* serotype 8 was isolated according to Saito & Miura [31] as follows: a 10 ml culture was grown until OD_600_ of 0.5 was reached, then pelleted and resuspended in 200 µl TE buffer (pH 8) containing 25% w/v sucrose, 60 mM EDTA, 1.6% SDS and 12.5 µg proteinase K. The suspension was incubated for 16 h at 37°C until a clear lysate was obtained, then the debris was removed by centrifugation at 3500*g* for 5 min. The supernatant was extracted with phenol then separated from contaminating proteins using successive washes with chloroform : isoamyl alcohol 24 : 1 until no protein was seen at the interface between organic and aqueous phases. DNA was precipitated using 2.5 volumes 100% ethanol and 0.1 volumes 3 M sodium acetate (pH 5.2), washed with 70% ethanol and resuspended in MilliQ water.

The capsule locus of serotype 8 was amplified from the genomic DNA using primers SP8F (5′-ATAATCTGCAGGGGAGTTGTGTTGAATAAATTCG) and AliAR (5′-TAATCTGCAGACTGCCGCGTATTCTTCACC) and Accuprime *Taq* HiFi (Invitrogen, UK) with the conditions: 95°C 30 s, 50°C 30 s, 68°C 9 min for 30 cycles.

Synthesized constructs or PCR-amplified fragments were then subcloned into vector pBBR1MCS-3 [32] using a protocol modified from Osoegawa *et al*. [33]. Briefly, inserts or vectors were digested with appropriate restriction enzymes. Where necessary, cut vector was treated with Antarctic phosphatase (NEB). Cut insert and vector were separated by agarose gel electrophoresis, DNA was recovered via electroelution and running buffer removed via dialysis. Impurities were removed using phenol chloroform extraction and DNA precipitated using 2.5 volumes ethanol and 0.1 volumes 3 M sodium acetate (pH 5.2). Insert and vector were ligated using T3 DNA ligase (NEB) at a molar ratio of 3 : 1. The ligation mixture was dialysed against MilliQ ultrapure water before transforming DH10B electrocompetent cells (NEB) in a 0.1 cm gap cuvette at 2 kV, 200 Ω and 25 µF.

### 2.3. Expression of capsule and immunoblot

*Escherichia coli* W3110 [34] cultures grown for 16 h were diluted into fresh SSOB media to an OD_600_ of 0.03. The media was supplemented with 1 mM IPTG and 4 mM MnCl_2_ and incubated at 28°C for 24 h. All samples were OD_600_ matched, washed with PBS and then lysed using a Bioruptor ultrasonic processor (Diagenode, Belgium) set on a high pulse rate set for 30 s on 30 s off for 15 min. Lysed samples were mixed with SDS–PAGE sample buffer and separated on 10% bis–tris gels in MOPS buffer (Invitrogen). Samples were electroblotted onto nitrocellulose membrane (GE Healthcare) using a semi-dry transfer unit (AA Hoefer). Membranes were blocked for 1 h in PBS containing 2% w/v skimmed milk powder. Serotype appropriate rabbit anti-capsule antibody (Statens Serum Institut, Denmark) was used at a dilution of 1 : 1000 in PBS containing 2% w/v skimmed milk powder and 0.1% v/v Tween 20. After 1 h incubation with primary antibody, membranes were washed three times with PBS (0.1% Tween 20) and then incubated for 45 min with a secondary goat anti-rabbit IgG IRDye800 conjugate antibody at a dilution of 1 : 10 000. Membranes were washed a further three times in PBS (0.1% Tween 20) and once with PBS before signal detection with the Odyssey LI-COR detection system (LI-COR Biosciences UK Ltd).

### 2.4. Immunofluorescence staining and microscopy

Induced *E. coli* cultures as described above were washed with PBS, and then 15 µl of cell suspension was air-dried on a glass coverslip before heat fixing. The coverslip was washed in PBS to remove unfixed cells before blocking in PBS containing 5% v/v foetal calf serum (FCS) for 1 h. After washing three times with PBS, coverslips were incubated with serotype appropriate rabbit anti-capsule antibody (Statens Serum Institut, Denmark) at a dilution of 1 : 1000 in PBS 5% FCS for 1 h. Following another three washes with PBS, the coverslips were incubated with Alexa Fluor 488 goat anti-rabbit IgG antibody conjugate (Life Technologies Ltd., UK) 1 : 1000 in PBS 5% FCS for 45 min. The coverslips were washed three times with PBS and allowed to air dry before mounting with SlowFade Gold antifade Mountant with DNA-specific counter stain 4′,6-diamidino-2-phenylindole (Life Technologies Ltd., UK). Each slide was visualized under fluorescence using an Olympus FluoView laser scanning microscope (Olympus Imaging and Audio Ltd.)

### 2.5. Indirect enzyme-linked immunosorbent assay

*Escherichia coli* cultures were grown and induced as described above. *S. pneumoniae* cultures were grown to CFU ml^−1^ of roughly 2 × 10^8^. Colony forming units (CFUs) were determined for all cultures in duplicate. Cells were washed in PBS and sonicated prior to coating a MaxiSorp microtitre plate (Nunc, UK) overnight at 4°C. A standard curve was generated using dilutions of purified pneumococcal polysaccharide (Statens Serum Institut, Denmark). Samples were blocked with PBS containing 5% milk for 2 h, followed by incubation with anti-capsule antisera (Statens Serum Institut, Denmark) at a dilution of 1 : 1000 in PBS 5% milk for 1.5 h. After washing with PBS 0.05% Tween, goat anti-rabbit IgG HRP (Abcam, UK) was added at a dilution of 1 : 120 000 in PBS 5% milk for 1 h. After washing with PBS 0.05% Tween, TMB (eBioscience, UK) was added, and the reaction was stopped with 2M H_2_SO_4_. Indirect ELISA detection was performed using a Dynex MRX II 96-well plate reader at an absorbance of 450 nm. Data analysis and graphing were performed using GraphPad Prism version 6.04 for Windows, GraphPad Software, La Jolla, CA (www.graphpad.com). All experiments represent three biological replicates, with each experiment performed in duplicate. Values were expressed as means ± standard errors of the mean. Variable were compared for significance using a Kruskal–Wallis test by ranks followed by a Dunn's test between pairs.

### 2.6. Lipopolysaccharide extraction and silver stain

*Escherichia coli* cultures containing recombinant or empty plasmid controls were grown and induced as described above. Purification of lipopolysaccharide (LPS) was carried out as described previously [35] using 100 mg of culture pellet for each sample. Samples were separated on a 4–12% bis–tris polyacrylamide gel using MES buffer. The gel was stained using a SilverQuest silver staining kit (Life Technologies Ltd., UK), according to the manufacturer's instructions, and visualized using a GeneGenius bioimaging system (Syngene, UK).

### 2.7. Mutagenesis by transposon insertion

The EZ-Tn5 <KAN-2> Insertion kit (Epicentre, USA) was used according to the manufacturer's protocol to disrupt genes within the cloned capsular loci. Briefly, 0.2 µg of plasmid DNA was mixed with reaction buffer, a molar equivalent of EZ-Tn5 <KAN-2> transposon and transposase, in a total reaction volume of 10 µl. Reactions were incubated at 37°C for 2 h before stop solution was added and the reaction incubated for a further 10 min at 70°C. The mutated plasmid DNA was transformed into electrocompetent DH10B *E. coli* cells (NEB) and cultured on plates containing 50 µg ml^−1^ kanamycin. Resulting kanamycin resistant clones were screened for mutation by a combination of PCR and sequencing.

## 3. Results

### 3.1. Design of constructs

**Figure 1.**
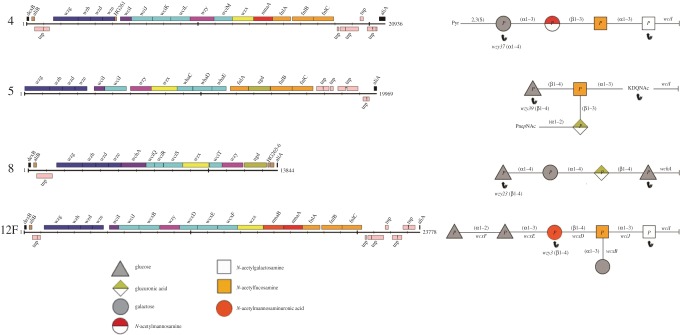
*Streptococcus pneumoniae* capsular polysaccharide loci and structures. The capsule coding loci, between *dexB* and *aliA*, for serotypes 4, 5, 8 and 12F are shown. Genes are represented by coloured boxes. Repeat units are shown with the reducing end sugar at the right-hand side. Monosaccharides are represented by coloured shapes according to the structure key. Polymerizing linkages (i.e the linkage between single subunits to form a polymer) are indicated by black arrows. Reproduced with permission from Bentley *et al.* [28].

Four serotypes that represented a variety of different structural properties and cloning challenges were selected for expression within *E. coli*. The sizes ranged from 9 to 16 kb encoding between four and six structural components, with or without side branches (figure 1). It is unlikely that synthase-dependent capsules would be substrates for the oligosaccharyl transferase PglB, used in PGCT, as they are built on a phosphatidylglycerol carrier rather than undecaprenol pyrophosphate, the universal glycan lipid carrier [36]. Therefore, we focused on Wzy-dependent capsule structures that comprise 93 out 95 serotypes.

**Figure 2.**
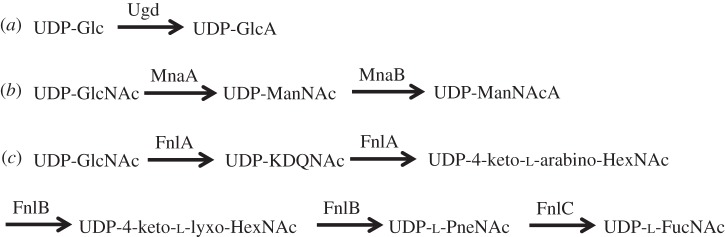
Biosynthetic pathways of *S. pneumoniae* encoded on the capsule loci. Ugd is a UDP-glucose 6 hydrogenase; MnaA is a UDP-*N*-acetylglucosamine-2-epimerase; MnaB is a UDP-*N*-acetylmannosamine dehydrogenase. FnlA–FnlC perform multiple steps in the synthesis of UDP-FucNAc. The first intermediate produced by FnlA, UDP-KDQNAc, and the second intermediate produced by FnlB, UDP-PneNAc, are both used in the capsule structure of serotype 5 in addition to UDP-FucNAc [30]. This is unique among the capsule structures identified for serotypes that contain the *fnl* locus.

The serotypes chosen were predicted to contain all the necessary genes within the locus and/or provided by *E. coli* central metabolism. For the pathways encoded within the locus, the precursor substrates are present (figure 2). Serotype 8 was selected as it contains only four sugars per repeat unit (two glucose units, galactose and glucuronic acid) and is polymerized in a linear fashion, with a bond formed between the first and last sugars (figure 1). There are no side branches, and there are predicted functions for all genes, two of which have been experimentally verified: *wciS* [37] and *wchA* [38]. A 9 kb amplicon is predicted to incorporate all the genes necessary for expression in *E. coli*; UDP-glucose and UDP-galactopyranose are present within *E. coli* K12, and glucuronic acid can be synthesized via Ugd from UDP-glucose.

Serotype 4 was selected as it contains an acetylated reducing end sugar so is predicted to be a substrate for PglB oligosaccharyltransferase [39]. The capsule contains four sugars (*N*-acetylgalactosamine, *N*-acetylfucosamine, *N*-acetylmannosamine and galactose), with a pyruvate attached to the fourth sugar, galactose (figure 1). Pyruvate can be provided from phosphoenolpyruvate, which is a product of glycolysis in *E. coli*. There are no side branches, and the polymerizing linkage occurs between the first and the fourth sugar. A 14 kb amplicon is predicted to contain all the necessary genes to express the capsule in *E. coli*; *N*-acetylglucosamine and galactose should be available in *E. coli*. The other two sugars, *N*-acetylfucosamine and *N*-acetylmannosamine, are synthesized from UDP-GlcNAc via FnlA-C and MnaA, respectively (figure 2).

Serotype 5 was selected as a further challenge for expression. The capsule repeat unit consists of five sugars, including an acetylated reducing end sugar and a side branch of two sugars (figure 1). The serotype 5 capsule is unusual in the fact that it is the only known capsular structure to incorporate the intermediates of the *fnl* pathway, 4-keto-*N*-acetyl-d-quinovosamine and *N*-acetylpneumosamine, in addition to the end product *N*-acetylfucosamine. This provides a particular challenge as it is uncertain whether these intermediates will be present in sufficient quantity to assemble the capsule in *E. coli*. The other two sugars in the capsule are glucose and glucuronic acid which as discussed above should be available in *E. coli* through central metabolism or the Ugd enzyme, respectively. It is predicted that a 13.5 kb region contains all the elements necessary for recombinant expression.

Serotype 12F was the most challenging serotype selected. This is the largest capsule region attempted at 16 kb which presents a significant cloning challenge. In addition, the capsule has an unusual secondary structure containing six sugars, a side branch and polymerizing linkage between first and third sugars (figure 1). The constituent sugars are two units of glucose, and one each of galactose, *N*-acetylfucosamine, *N*-acetylmannosaminuronic acid and *N*-acetylgalactosamine at the reducing end. Serotype 12F uses only the end product of the *fnl* pathway, *N*-acetylfucosamine, as in serotype 4. Unlike serotype 4, serotype 12F uses both MnaA and MnaB to produce *N*-acetylmannosaminuronic acid from UDP-GlcNAc.

It was decided that to preserve gene expression, the locus should be cloned into a single plasmid. Early attempts to clone the locus including the regulatory genes, *wzg–wze,* failed (data not shown). The role of regulatory genes, *wzg–wze*, in *S. pneumoniae* is not fully understood, but each are necessary for capsule production and export of capsule in the native organism [40]. However, these genes do not encode structural features, so may not be required for expression of the capsule in *E. coli*.

### 3.2. Testing recombinant expression

In the capsular polysaccharide encoding loci, it is not known whether a native promoter may be present downstream of *wzg–wze*. There is thought to be one promoter upstream of *wzg* (*cpsA*), and the entire locus is transcribed as a single operon [41]. For serotypes 4, 5 and 12F, there is a gap between these regulatory genes and the initial transferase *wciI*; for serotype 8 there is no such gap. In all cases, some sequence upstream of the initial transferase was incorporated into the recombinant locus in case there were regulatory elements present. The upstream sequence was scanned for stem loops that could denote rho-independent terminators. The locus was subcloned into the vector pBBR1MCS-3 for expression; a DHFR cassette was included at the 3′ end of constructs for serotype 5 and serotype 12F to aid with vector switching. Once cloned into the pBBR1MCS-3 vector, the construct was verified by sequencing and was then transformed into *E. coli* W3110 cells for expression testing.

**Figure 3.**
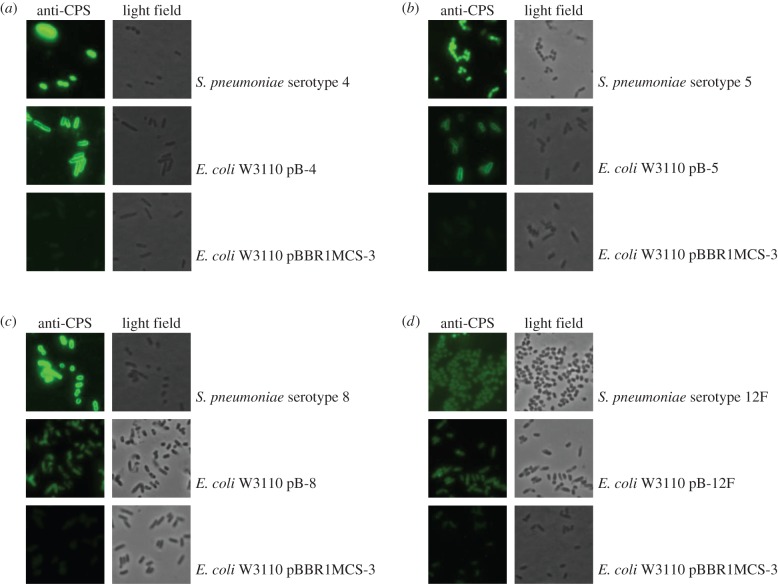
Immunofluorescence microscopy of recombinantly expressed *S. pneumoniae* capsule loci. *E. coli* cells carrying the capsule expression vectors (*a*) pB-4, (*b*) pB-5, (*c*) pB-8 and (*d*) pB-12F were probed with group or type-specific anti-capsular antibody and Alexa Fluor 488 conjugated secondary antibody. Empty vector *E. coli* and *S. pneumoniae* of relevant serotype were used as controls. Images are shown at 100× magnification.

To test for expression and location of recombinantly expressed polysaccharide, cells were probed with capsule type or group-specific antisera and subjected to immunofluorescence microscopy (figure 3). Expressed glycan can be seen coating the surface of cells in the O-antigen ligase positive *E. coli* strain, W3110. The level of fluorescence observed for the strains expressing recombinant polysaccharide is higher than for the empty vector control (figure 3), although low level cross-reactivity with the *E. coli* cells may be seen for all serotype antisera.

**Figure 4.**
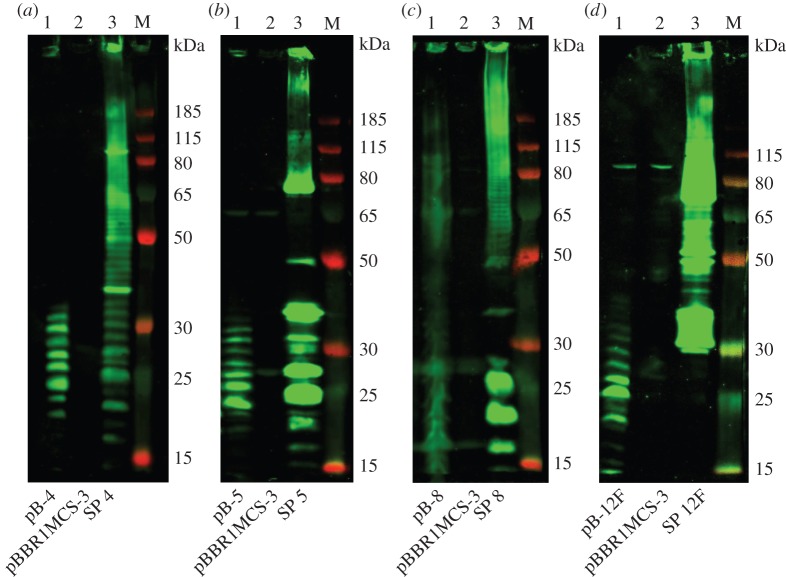
Immunoblot of recombinantly expressed *S. pneumoniae* capsule in *E. coli.* Lysed, whole cell samples were separated by SDS–PAGE on a 10% bis–tris gel and detected using anti-serotype CPS primary antibody and anti-rabbit fluorescent secondary antibody. (*a*) Serotype 4; (*b*) serotype 5; (*c*) serotype 8; and (*d*) serotype 12F. Lane 1: *E. coli* W3110 carrying recombinant plasmid; lane 2: *E. coli* W3110 carrying empty vector pBBR1MCS-3; lane 3: *S. pneumoniae* wild-type capsule. M, molecular weight marker PageRuler Plus.

To test for polymerization of the capsular polysaccharide, *E. coli* W3110 cells containing the recombinant expression plasmids, along with empty vector controls, were induced and grown for 24 h before lysing. Lysates were analysed for capsule-specific expression by SDS–PAGE and immunoblot (figure 4). A ladder-like pattern is clearly observed for all samples expressing recombinant polysaccharide, and is absent from the empty plasmid control. It is likely that this ladder-like pattern represents different chain lengths of the capsule repeat unit. The recombinant serotype 8 plasmid is the only one that shows high molecular weight polymerization and a banding pattern similar to the wild-type. For the *S. pneumoniae* samples, a high molecular weight smear can be seen along with distinct bands in a ladder-like pattern towards the lower molecular weight marker bands. The capsule from wild-type serotypes 5 and 12F do not run in a ladder pattern. It has been demonstrated previously that the rate of migration through a gel of polysaccharides is dependent not only on molecular mass but also on charge and secondary structure [42]. As serotypes 5 and 12F contain side branches this may lead them to have a more complicated secondary structure and thus impact on their migration through the gel. For the recombinant polysaccharides, the banding pattern is not equivalent to the wild-type polysaccharides; this may be indicative of the fact that the level of polymerization for the recombinantly expressed polysaccharide is not as high as in the native organism, *S. pneumoniae*.

**Figure 5.**
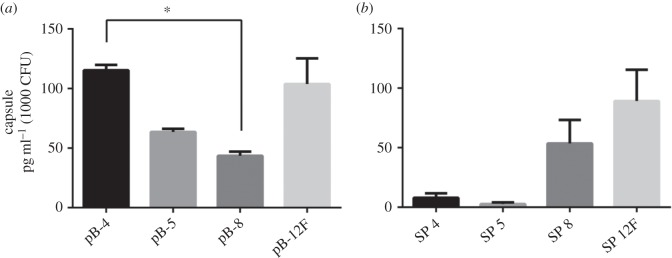
Quantification of capsular polysaccharide production by ELISA. Biological triplicate samples were processed in duplicate. Values for amount of capsule produced were interpolated from a standard curve of purified type-specific capsular polysaccharide (Statens Serum Institut, Denmark) and expressed as capsule produced per 1000 CFU (calculated from duplicate plating of each sample). Data are presented as the mean of biological replicates with error bars denoting the standard error of the mean. Significance was determined using a Kruskall–Wallis test by ranks followed by a Dunn's test between pairs. *p < 0.05. (*a*) Recombinant polysaccharide expression in *E. coli*: pB-4 values were an average of 115 pg ml^−1^ (1000 CFU); pB-5 values were an average of 64 pg ml^−1^ (1000 CFU); pB-8 values were an average of 44 pg ml^−1^ (1000 CFU); and pB-12F values were an average of 104 pg ml^−1^ (1000 CFU). (*b*) Polysaccharide expression from *S. pneumoniae*. SP 4 values were an average of 8 ng ml^−1^ (1000 CFU); SP 5 values were an average of 3 ng ml^−1^ (1000 CFU); SP 8 values were an average of 54 ng ml^−1^ (1000 CFU); and SP 12F values were an average of 89 ng ml^−1^ (1000 CFU).

To quantify expression between the recombinant strains and to assess the expression levels compared with the wild-type organism, indirect ELISA was performed (figure 5). Recombinant serotype 4 capsule expression was higher than for the other recombinant polysaccharides, although the difference was only significant between pB-4 and pB-8. There was a high degree of variability between different colonies expressing pB-12F. The average expression levels varied between 44 pg ml^−1^ for 1000 CFU of pB-8 and 115 pg ml^−1^ for 1000 CFU of pB-4. The levels of recombinant expression were around 100-fold less for pB-4 and pB-5, and 1000-fold less for pB-8 and pB-12F. However, the levels of capsular polysaccharide expression for *S. pneumoniae* were very variable with no statistically significant difference between the serotypes. The capsule of *S. pneumoniae* is known to be phase variable [43] and to impart a fitness cost when grown *in vitro* [14], which may be responsible for some of the variation seen.

### 3.3. Identifying genes necessary for recombinant expression

**Figure 6.**
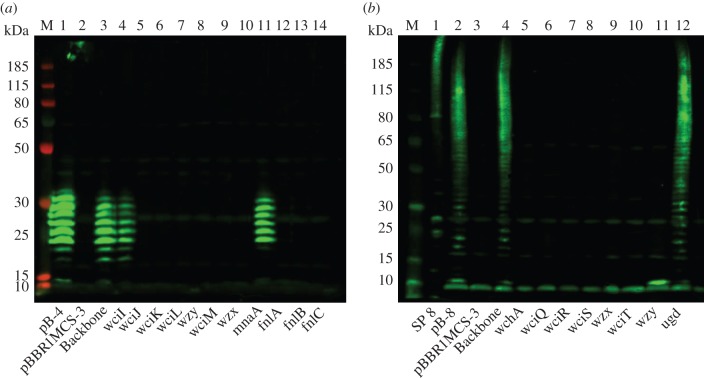
Immunoblots show polysaccharide production from transposon mutants. Transposon mutants were selected in each of the cloned genes in the polysaccharide encoding locus. (*a*) Serotype 4 and (*b*) serotype 8. Lysed, whole cell samples were separated by SDS–PAGE on a 10% bis–tris gel and detected using anti-serotype CPS primary antibody and anti-rabbit fluorescent secondary antibody. pB-4 (A1) and pB-8 (B2) are the original plasmids containing no transposon insertion for comparison. Empty vector (A2, B3) and transposon insertion in the plasmid backbone (A3, B4) are included as negative and positive control respectively. SP 8 (B1) is *S. pneumoniae* serotype 8 capsule expressed from the wild-type organism. M, molecular weight marker PageRuler Plus. Lanes (*a*) 4–14 and (*b*) 5–B12 represent transposon mutants in the genes indicated in the order they appear in the capsule-encoding operon.

In order to determine whether it is necessary to heterologously express genes for sugar precursors found in *E. coli* that are also located in the *S. pneumoniae* capsule locus, saturation transposon mutagenesis of the capsule-encoding plasmids, pB-4 and pB-8, was undertaken. The EZ-Tn5 <KAN-2> transposon was chosen as it contains no transcriptional terminators and is therefore less likely to produce significant negative polar effects upon insertion. It was hoped that mutagenesis would confirm the expected role of some of the genes and provide an idea of the minimum gene set required to express the capsule epitope. Transposon insertion sites are shown in the electronic supplementary material, figure S1. The mutated recombinant expression plasmids were induced and cells were grown for 24 h before lysis and analysis by SDS–PAGE and immunoblot with serotype-specific antisera (figure 6). As expected, a transposon insertion within the plasmid backbone had no effect on capsule expression. The majority of transposon insertions within the coding regions in the loci of serotypes 4 and 8 resulted in a loss of detectable epitope as visualized by lack of antiserum binding in the immunoblot. The exceptions are *mnaA* in serotype 4 and *ugd* in serotype 8, whereby no difference in antiserum reactive polymer is seen between the original and mutated plasmids. In addition, in serotype 4, the initial transferase *wciI* can be mutated without disrupting polymer production in this strain of *E. coli*. When the polymerase, *wzy*, of serotype 8 is mutated a bright antiserum reactive band is visible at the base of the gel (at around 15 kDa) that is absent from the empty vector control lane, possibly indicating a single repeat unit of the capsule attached to lipid A.

### 3.4. Expressing serotype 4 capsule in different strains of *Escherichia coli*

**Figure 7.**
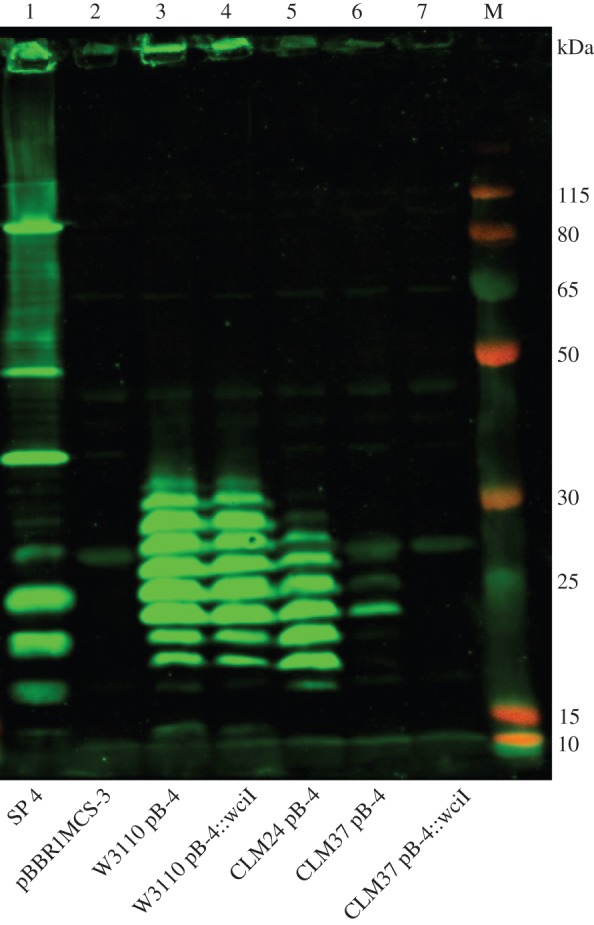
Immunoblot of recombinant *S. pneumoniae* serotype 4 capsule in different strains of *E. coli*. Lysed, whole cell samples were separated by SDS–PAGE on a 10% bis–tris gel and detected using anti-serotype CPS primary antibody and anti-rabbit fluorescent secondary antibody. Lane 1: *S. pneumoniae* serotype 4; lane 2: *E. coli* W3110 carrying empty vector pBBR1MCS3; lane 3: *E. coli* W3110 carrying recombinant pB-4 plasmid; lane 4: *E. coli* W3110 carrying recombinant pB-4 plasmid with a transposon insertion in initial transferase *wciI*; lane 5: *E. coli* CLM24 (ΔwaaL) carrying recombinant pB-4 plasmid; lane 6: *E. coli* CLM37 carrying recombinant pB-4 plasmid; lane 7: *E. coli* CLM37 (ΔwecA) carrying recombinant pB-4 plasmid with a transposon insertion in initial transferase *wciI.* M, molecular weight marker PageRuler Plus.

**Figure 8.**
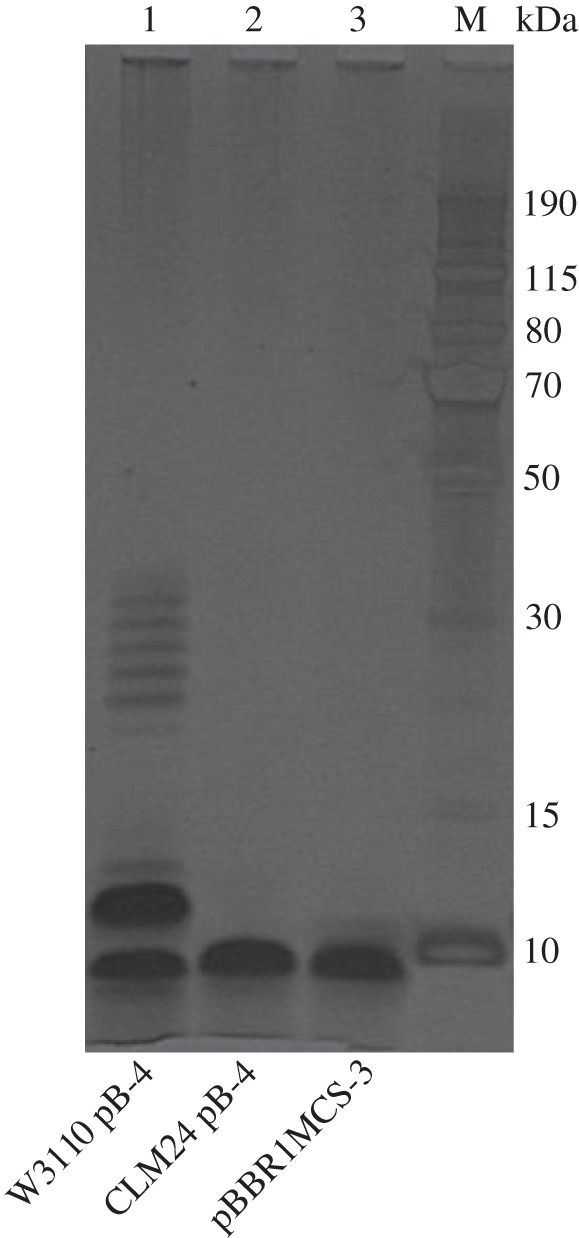
Silver stain of *E. coli* LPS extracts for recombinantly expressed *S. pneumoniae* serotype 4 capsule*.* LPS was extracted using hot phenol from 100 mg of induced culture pellet. Samples were separated on a 4–12% bis–tris gel using MES running buffer. The gel was subsequently silver stained using a SilverQuest Silver staining kit (Life Technologies, UK). Lane 1: *E. coli* W3110 carrying recombinant pB-4 plasmid; lane 2: *E. coli* CLM24 (ΔwaaL) carrying recombinant pB-4 plasmid; lane 3: *E. coli* W3110 carrying empty vector pBBR1MCS3. M, molecular weight marker PageRuler Plus.

To assess the effect of native *E. coli* genes on recombinant production of capsular polysaccharide, pB-4 was expressed in different strains of *E. coli.* *E. coli* CLM24, an *E. coli* W3110 derivative with the *O-*antigen ligase, *waaL,* deleted [20], was transformed with the pB-4 plasmid, to explore whether the ligase is competing with the recombinantly expressed polymerase, Wzy. *E. coli* CLM37, an *E. coli* W3110 derivative with the initial transferase for the enterobacterial common antigen (ECA), *wecA,* deleted [44], was also transformed with pB-4. To explore whether WecA could be compensating for the absence of the initial transferase, WciI, from the *S. pneumoniae* serotype 4 capsule, pB-4 with a transposon insertion in *wciI* was also transformed into CLM37. All strains containing recombinant plasmids or empty vector controls were induced and grown for 24 h before lysing. Lysates were analysed for expression of the serotype 4 capsule by SDS–PAGE and immunoblot (figure 7). Lanes 3 and 4 show that when *wciI* is mutated, recombinant polysaccharide is still produced to similar levels as from the un-mutated plasmid pB-4, when expressed in W3110 *E. coli.* There is very little polysaccharide produced in the CLM37 strain (lane 6), and production is abolished in the *wciI* mutant (lane 7), suggesting that WecA from *E. coli* is indeed compensating for the *S. pneumoniae* WciI in this context. In the ligase negative strain, CLM24 (lane 5), recombinant polysaccharide is produced but with a banding pattern that does not extend to the equivalent higher protein marker bands as that produced in W3110. In addition, there is an absence of an antiserum reactive band that runs equivalent to the 15 kDa marker band in CLM24 when compared with the polysaccharide produced in W3110. This possibly denotes that, in W3110, the shift seen is due to the attachment to lipid A, which is a larger molecule than undecaprenol. In order to assess the contribution of WaaL on mobility of the polysaccharide through the gel, LPS extractions followed by SDS–PAGE and silver staining were carried out (figure 8). A ladder-like banding pattern is only observed in strain W3110 carrying the recombinant plasmid pB-4, suggesting that WaaL is indeed attaching the polysaccharide to the lipid A core. This is not seen when the recombinant capsule is expressed in CLM24 which lacks WaaL. This strain has a pattern identical to that of W3110 carrying an empty plasmid as a control.

## 4. Discussion

The cloning and stable expression of complex polysaccharides in *E. coli* is important for synthetic glycobiological development. Which genes are directly involved from imported loci and which genes are compensated for by the *E. coli* host strain has been poorly studied. Recombinant polysaccharides can be used in vaccine production; indeed, there are many recent examples of using *E. coli* as a work horse for vaccine production against pathogens such as *Francisella tularensis* [21], *Burkholderia pseudomallei* [22], *Shigella flexneri* 2a [23] and the Gram-positive *Staphylococcus aureus* [24]. Using PGCT circumvents the need to use chemical conjugation, which is expensive, multi-step and creates heterogeneous linkage species [19]. The first step in the process of making such conjugate vaccines is to reliably recombinantly express the polysaccharide of interest within *E. coli.* In addition, producing the polysaccharide within *E. coli* would eliminate the need to grow pathogenic organisms for vaccine production. For *S. pneumoniae*, the full recombinant expression of capsular polysaccharides would be of significant value towards the low cost generation of glycoconjugate vaccines that have been proven to be effective at protecting against a globally important bacterial pathogen.

Alternative vaccine strategies such as live cell delivery and outer membrane vesicles (OMVs) have been explored for a variety of pathogens. For example, live avirulent *Salmonella* have been proposed as vectors for delivery of *S. pneumoniae* proteins, conferring protection in mice [45]. *S. pneumoniae* Rx1 unencapsulated strain, with *lytA* mutation and pneumolysin toxoid, adsorbed with aluminium hydroxide (pneumococcal whole cell vaccine, wcv), conferred immunity to different strains when used to vaccinate mice [46]. Wcv has been shown to be amenable to scaled up production according to current good manufacturing process regulations [47] and is currently in phase 2 clinical trials [48]. OMVs have been used for vaccination against an epidemic strain of *Neisseria meningitidis* serogroup B, in New Zealand [49]. *Salmonella enterica* serovar Typhimurium OMVs delivering pneumococcal surface protein, PspA, have been shown to protect against challenge with *S. pneumoniae* in mice [50]. It is possible that OMVs, including those from *E. coli,* could be used to deliver recombinant polysaccharide.

New vaccine production methods are needed for *S. pneumoniae* as changes in commonly isolated serotypes change geographically and over time, especially after the introduction of vaccines. A surveillance study covering 26 European countries in 2010 found that, after the introduction of PCV7, the most commonly isolated serotypes were 19A, 1, 7F, 3, 14, 22F, 8, 4, 12F and 19F [51]. The four most commonly isolated serotypes were not present in PCV7, suggesting serotype replacement. A large increase in the incidence of serotype 19A was also observed in the USA after introduction of PCV7 [52]. Serotypes 1, 3, 5, 6A, 7F and 19A were subsequently added into the formulation of PCV13. Vaccination strategies have been largely successful with a reduction in disease also among non-vaccinated individuals. However, an increase in non-vaccine serotypes has been observed, especially in those under 5 and over 45 years, with serotypes 12F, 8, 15A and 24F increasing significantly in the UK, after introduction of PCV13 [53].

In this study, four different capsule structures have been expressed within *E. coli.* Little is known about the regulation of the capsule in *S. pneumoniae;* it is thought that the capsule is transcribed as a single operon based on studies with serotype 19F, from a promoter upstream of *wzg* [25,41]. The amount of capsule produced has been proposed to be regulated at the post-translational level via a multiprotein phosphorelay system encoded by *wzg–wze* (*cpsA–D*) [40,54,55]. The recombinant loci in this study did not contain these four regulatory genes, or the proposed native capsule promoter, and yet polysaccharides that showed reactivity to serotype-specific antisera were still produced (figure 4). This suggests that within *E. coli* these genes are not necessary for recombinant expression of the *S. pneumoniae* capsular polysaccharide. From figure 4, it can be seen that only short polymers are present for all except serotype 8. There are several possible reasons for this, including: shortage of precursors, incorrect incorporation of sugars leading to premature termination of the polysaccharide chain, insufficient expression of polymerase or lack of chain length regulation. In *S. pneumoniae,* D39 short polymers have been seen when either *wzd* (*cpsC*) or *wze* (*cpsD*) are mutated, raising the possibility that these enzymes have a function in chain length regulation [55]. In *E. coli,* Wzc influences chain length; autophosphorylation is required for high molecular weight K30 capsular polysaccharide assembly [56]. Wzh (CpsB) from *S. pneumoniae* has been shown to dephosphorylate Wzc from *E. coli,* despite having a very different structure from Wzb, the native phosphatase of *E. coli* [57], indicating the possibility of cross-talk between species. It is unknown what effect native chain length regulators from *E. coli* may have on recombinant polysaccharide production. The polysaccharide chain length is likely to have an impact on vaccine efficacy. In a study of group B *Streptococcus* conjugates, an intermediate capsule size was found to produce optimal-specific protective antibodies [58]. The reason was proposed as a trade-off between the greater T-cell-dependent response of shorter chain lengths and the higher degree of expression of conformational epitope in longer chain lengths. Antibodies against *S. pneumoniae* serotype 14 showed lower affinity to shorter chain lengths, suggesting the importance of a conformational epitope [59]. Chain length was shown to have an impact on the optimal ratio of protein to saccharide for a pneumococcal type 4 conjugate vaccine, with saccharides of 12 repeating units requiring a higher ratio of saccharide per protein to elicit a protective response [60].

The recombinant loci for capsule expression in this study ranged in size from 9 to 16 kb. This represents a substantial amount of extraneous genetic material to replicate and genes to be expressed, which could place a burden on the *E. coli* host, not to mention the difficulty in manipulating large DNA fragments for subcloning into alternative vectors. It may not be essential to recombinantly express all the capsule sugar biosynthesis genes in *E. coli* if the substrates are already available. To explore this possibility, a transposon library was created and screened for insertions in each of the capsule locus genes, within the expression plasmid. The ability to produce the capsule epitope was then assessed. In the majority of cases, the transposon insertion mutations resulted in a lack of detectable epitope as can be seen in figure 6. As the flippase mutant also has no detectable levels of epitope, it is possible that the levels of recombinant capsule subunit produced are insufficient to generate a signal when still bound to Und-PP in the cytoplasm. Without the flippase function, the capsule repeat unit would not be released from Und-PP, thus not allowing for recycling of the carrier and further build up of the epitope. It also appears that none of the native *E. coli* flippases are capable of compensating for the function of recombinant *S. pneumoniae* Wzx. It was initially proposed that Wzx proteins have a relaxed specificity, only recognizing the first sugar of the polysaccharide subunit [61]. In *E. coli* K-12, it has been shown that the Wzx of the ECA can compensate for the Wzx of the O-antigen but only in the absence of its corresponding Wzy and Wzz proteins, suggesting that these proteins form a complex [62]. There are 13 homology groups for the *S. pneumoniae* Wzx flippases with no apparent link between homology group and initial sugar, so it is unclear how specific the flippase is for its corresponding capsule [30]. However, recent studies have shown that Wzx flippases show specificity for repeat unit structure in *Salmonella enterica* [63,64] and *E. coli* [65]. The Wzy polymerase knockout shows a lack of polymerized epitope. In figure 6*b* an antiserum reactive band can be seen at the base of the gel that is absent from the negative control, suggesting a single capsule unit. There are 40 homology groups for *S. pneumoniae* Wzy polymerases that appear to associate with initial transferase and linkage catalysed [30]. This result suggests that the *S. pneumoniae* polymerase is specific enough that no *E. coli* polymerase can compensate for its function, for these particular serotypes.

From figure 6, it can be seen that in the serotype 4 capsule, MnaA, a UDP-GlcNAc 2-epimerase that produces *N*-acetylmannosamine is compensated for by a native *E. coli* gene. *N*-acetylmannosamine is produced by MnaA from UDP-GlcNAc. RffE or WecB from the ECA biosynthesis pathway can perform this reaction in *E. coli*. *N*-acetylmannosamine is used as a substrate for conversion to *N*-acetylmannosaminuronic acid by MnaB. This reaction is also performed in *E. coli* by RffD or WecC. UDP-ManNAc (*N*-acetylmannosamine) is a more common capsule component, found in nine out of the 54 known *S. pneumoniae* capsule structures, whereas UDP-ManNAcA (*N*-acetylmannosaminuronic acid) is found in only two out of the 54 known capsule structures. There are no known examples of *S. pneumoniae* capsules that incorporate both UDP-ManNAc and UDP-ManNAcA. If MnaA is not needed for recombinant expression of serotype 4 capsular polysaccharide, then it is possible that MnaA and MnaB could be removed from the recombinant 12F capsule locus without affecting expression. From figure 6, it can also be seen that in the serotype 8 capsule, Ugd, a UDP-glucose 6-dehydrogenase producing glucuronic acid, can be compensated for in *E. coli*. UDP-GlcA (glucuronic acid) can be produced using Ugd from UDP-Glc; Ugd is also present in *E. coli*. Glucuronic acid is present in 11 of the 54 known *S. pneumoniae* capsule structures, including serotype 5. Others have reported the expression of Gram-positive capsules in *E. coli* by using biosynthesis genes from different sources to increase efficiency of expression [24]. *Staphylococcus aureus* type 5 and type 8 capsular polysaccharide were reproduced by expressing both *S. aureus* and *Pseudomonas aeruginosa* genes, with biosynthesis of ManNAcA produced by native *E. coli* pathways [24].

Also from figure 6, it can be seen that none of the enzymes in the *fnl* pathway can be compensated for in *E. coli* W3110*.* FnlA, FnlB and FnlC produce UDP-KDQNAc, UDP-PneNAc and UDP-l-FucNAc, respectively, from a UDP-GlcNAc precursor. UDP-l-FucNAc is found in five of the 54 known *S. pneumoniae* structures. Only serotype 5 is known to use the intermediates in this pathway as components in the capsule. As shown in figure 2, the reducing end sugar KDQNAc is actually the product of the first reaction catalysed by FnlA from UDP-GlcNAc. It is unknown if the sequence divergence between *fnlA* genes in serotype 5 and e.g. serotype 4 would be enough to allow for a build up of the first intermediate for use in capsule generation [30]. FucNAc is also found as a component in ECA, but the precursor is TDP linked and 4-acetamido-4,6-dideoxy-d-galactose [66] as opposed to the UDP-2-acetamido-2,6-dideoxy-l-galactose required for *S. pneumoniae* capsule synthesis [30]. FnlA-C homologues are found in the O-antigen synthesis locus of *E. coli* 0172 but not in K12 derivatives [67].

An understanding of the biosynthesis pathways in *E. coli* is necessary to avoid potential cross-talk between native and exogenous pathways. *E. coli* uses undecaprenyl pyrophosphate as the lipid carrier for O-antigen, colanic acid and ECA assembly [36]. Each of these pathways has an initial transferase which may compete with heterologously expressed genes during recombinant capsule synthesis. From figure 5, it can be seen that in serotype 4, *E. coli* W3110 can compensate for the initial transferase WciI, which in *S. pneumoniae* transfers *N*-acetylgalactosamine onto the lipid carrier. In the ECA biosynthesis pathway, WecA transfers *N*-acetylglucosamine onto the lipid carrier [66]. It has been proposed that WecA can substitute for the initial sugar transferase, PglC, in the *Campylobacter jejuni* heptasaccharide biosynthesis locus, where the initial sugar is bacillosamine. When the Pgl locus was recombinantly expressed in *E. coli,* a heptasaccharide was still produced when PglC was inactivated. In an *E. coli* strain lacking WecA, no recombinant glycan was observed in the PglC mutant [44]. From figure 7, it can be seen that the recombinant polysaccharide is still produced without the initial transferase WciI when expressed in a WecA positive *E. coli* strain, but not in a WecA negative strain. If WecA is indeed compensating for the initial transferase WciI that would suggest that in the *wciI* mutant the initial sugar is GlcNAc as opposed to GalNAc. As there is still polymerization of the glycan observed in the *wciI* mutant, this also suggests that the polymerase has somewhat relaxed specificity. In order to ascertain how faithfully the recombinant polysaccharide is produced compared with the wild-type capsule, structural data would need to be obtained. However, this appears to have little effect on the epitope recognized by the capsule type-specific antisera. The availability of UDP-GalNAc is unknown within *E. coli* W3110. From figure 7, it appears that the serotype 4 polysaccharide is poorly produced in the WecA negative *E. coli* strain, CLM37, suggesting that availability of UDP-GalNAc may be a limiting factor. *E. coli* produces GalE, a UDP-glucose-4-epimerase, and although GalE enzymes primarily interconvert glucose and galactose, some enzymes from this grouping have been shown to have relaxed specificity and may interconvert acetylated forms [68]. The GalE epimerase of *C. jejuni* has been shown to have UDP–*N*-acetylglucosamine-4-epimerase activity [69], so addition of this enzyme may improve recombinant polysaccharide yield for serotypes 4 and 12F. In addition to cross-talk between pathways, not all necessary precursors are available in all strains of *E. coli.* For example, UDP-galactopyranose is found only in *E. coli* K12 derivatives, and not BL21 derivatives, as the Gal operon is mutated in BL21 [70]. Therefore, it would not be possible to express serotypes 4, 5 or 12F in *E. coli* BL21.

There is a precedent for bacteria-sharing intermediates in glycan biosynthesis pathways [71]. Examples include short polysaccharides transferred onto at least seven proteins by PglL in *Acinetobacter baumanii* [72] and short capsule polymers transferred to LPS by WaaL in *E. coli* [73]. However, from figure 7, it can be seen that expressing the capsule recombinantly in a ligase negative strain, CLM24, has little effect on the amount of capsule produced or the extent of polymerization. This suggests that WaaL is not competing with the capsule polymerase in this instance. It has been shown previously that the O-antigen ligase, WaaL, can attach recombinantly expressed glycans onto the lipid A core [20,74] in O-antigen negative *E. coli* strains. Figure 8 shows that the recombinant serotype 4 capsule is indeed attached to lipid A when WaaL is present. The ladder-like pattern of banding is absent from the LPS sample when the recombinant capsule is expressed in CLM24, which lacks WaaL. This suggests that the ladder pattern observed in figure 7, from the whole cell lysate of CLM24 expressing recombinant serotype 4 capsule, is bound to undecaprenol, which could explain the shift apparent when pB-4 is expressed in W3110, as undecaprenol is a smaller molecule than lipid A. From figure 3, it can be seen that the recombinant glycan is exported to the surface of the cell as the antiserum binds to non-permeabilized *E. coli* cells in the WaaL positive W3110 strain. Having the recombinant polysaccharide attached to the outer membrane could be of use in whole cell or OMV vaccine preparations.

Data from this study reveal *S. pneumoniae* capsular polysaccharides from serotypes 4, 5, 8 and 12F were recombinantly expressed at levels of 44–115 pg ml^−1^ per 1000 CFU. This is roughly 100- to 1000-fold less than from *S. pneumoniae* grown *in vitro.* In the case of serotype 8, the locus for recombinant expression is only 9 kb, and there are four components in the repeat unit, all of which can be supplied by *E. coli* central metabolism. Serotype 8 was the simplest capsule structure attempted in this study and was the serotype that showed the most abundant and highly polymerized recombinant expression. The recombinant serotype 4 locus was larger at 14 kb, and the capsule repeat unit contains five components, four of which can be supplied by *E. coli* central metabolism, and it has no side branches. This serotype showed the highest level of consistent recombinant expression. The recombinant serotype 5 locus was of a similar size at 13.5 kb, but the repeat unit contains five components, only two of which can be supplied by *E. coli* central metabolism. Three of the components are intermediates of the same pathway that could represent a bottleneck. In addition, serotype 5 has a side branch of two sugars which presents a more complicated secondary structure. These factors could explain why the expression of this serotype capsule was less successful. The recombinant serotype 12F capsule was the most variably expressed. The 12F locus was the largest cloned at 16 Kb, contains six components in the repeat unit and, although all but one of these components could be supplied by *E. coli* central metabolism, has a side branch and an unusual secondary structure [30].

In this study, a subset of 4 of the 95 known serotypes of *S. pneumoniae* have been cloned and expressed in *E. coli*. None of the structures known to contain AATgal, choline or ribitol were attempted for recombinant expression as this would have involved adding additional pathways into the host strain, as these components are not available within *E. coli* K12 derivative strains. In addition, none of the capsule structures contain sugars that are variably O-acetylated as it is unknown how this would be reproduced in *E. coli.* In *S. pneumoniae*, the pattern of O-acetylation is not obviously correlated with the acetyltransferase homology group [30], and can be variable even between repeat units of the same serotype [75]. The O-acetyl groups of several serotypes have been found to be immunologically important [29,75] and are easily lost via oxidation [75]. It is unlikely that all *S. pneumoniae* serotype capsules would be suitable for recombinant expression in *E. coli* and identifying bottlenecks in precursor biosynthesis would be needed to produce more complex structures. However, the methods presented in this study could be broadly applicable to other polysaccharides, not just from *S. pneumoniae* but also from other important pathogens.

## 5. Conclusion

We have produced *S. pneumoniae* capsules recombinantly in *E. coli,* with varying degrees of success. The stable expression of complex polysaccharides in *E. coli* is of considerable biotechnological importance, but poorly developed. This study paves the way for optimization of these recombinantly produced glycans which could then provide a source of glycan for vaccination strategies. The recombinant capsule would be suitable for both chemical and biological conjugation, providing an unlimited source of safely produced glycan that does not depend on growing the pathogen *S. pneumoniae.* We report expression of the capsular polysaccharide from serotypes 4, 8, 5 and 12F. In addition, this study has provided insights into rational design for recombinant expression and which further serotypes would be tractable for recombinant expression.

## Supplementary Material

Supplementary information
