# Supplementary material for: Recombinant expression of Streptococcus pneumoniae capsular polysaccharides in Escherichia coli
Source: Open Biol. 2016 Apr 13;6(4):150243. doi: 10.1098/rsob.150243 (PMC4838161; doi:10.1098/rsob.150243)
Supplement: Supplementary information [file rsob150243supp1.pdf]

Table S1: Strains and plasmids used in this study

Strain/ plasmid	Description	Source/ reference
E. coli DH10B	Δ(ara-leu) 7697 araD139 fhuA ΔlacX74 galK16 galE15 e14- Φ80dlacZΔM15 recA1 relA1 endA1 nupG rpsL (StrR) rph spoT1 Δ(mrr-hsdRMS-mcrBC)	NEB, UK
E. coli W3110	F- lambda- IN(rrnD-rrnE)1 rph-1	(Bachmann 1996)
E. coli CLM24	W3110 with the O-antigen ligase, *waaL,* deleted	(Feldman et al., 2005)
E. coli CLM37	W3110 with the initial transferase from ECA, *wecA,* deleted	(Linton et al., 2005)
S. pneumoniae strain 600/62	Serotype 4 strain	Statens Serum Insitut, Denmark
S. pneumoniae strain Ambrose	Serotype 5 strain	Statens Serum Insitut, Denmark
S. pneumoniae strain 573/62	Serotype 8 strain	Statens Serum Insitut, Denmark
S. pneumoniae strain 6312	Serotype 12F strain	Statens Serum Insitut, Denmark
pBBR1MCS-3	Cloning vector, Tet^R	(Kovach et al., 1995)
pB-4	pBBR1MCS-3 containing a 14095 bp region including *wciI-fnlC*, from *S. pneumoniae* serotype 4, synthesised by Epoch Life Sciences, cloned into KpnI XbaI sites of the MCS. Tet^R	This study
pB-5	pBBR1MCS-3 containing a 13506 bp region including *wciI-fnlC*, from *S. pneumoniae* serotype 5, synthesised by GeneWiz, cloned into ApaI site of the MCS. Tet^R $Trim^R$	This study
pB-8	pBBR1MCS-3 containing a 9085 bp region including *wchA-aliA*, from *S. pneumoniae* serotype 8, amplified by PCR, cloned into PstI site of the MCS. Tet^R	This study
pB-12F	pBBR1MCS-3 containing a 16158 bp region including *wciI-fnlC*, from *S. pneumoniae* serotype 12F, synthesised by GenScript, cloned into PstI site of the MCS. Tet^R $Trim^R$	This study

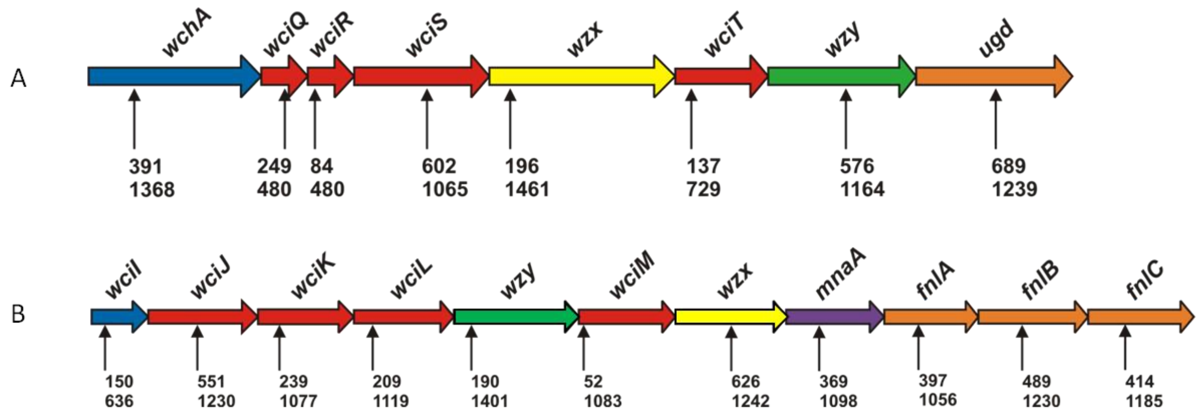

Supplementary Fig 1. Location of transposon insertion sites within the cloned capsule locus. A) Transposon location in pB-8, the cloned serotype 8 locus. B) Transposon location in pB-4, the cloned serotype 4 locus. Genes are represented by coloured arrows: undecaprenyltransferase, blue; glycosyltransferases, red; flippase, yellow; polymerase, green; and sugar biosynthesis enzymes, purple or orange. The approximate location of insertion of the transposon is indicated by the vertical black arrows; the top number indicates the insertion site of the transposon in bp from 5` end of the gene, the length of which is shown by the bottom number in bp.
